# Point-of-care, multispectral, smartphone-based dermascopes for dermal lesion screening and erythema monitoring

**DOI:** 10.1117/1.JBO.25.6.066004

**Published:** 2020-06-23

**Authors:** Ross D. Uthoff, Bofan Song, Melody Maarouf, Vivian Shi, Rongguang Liang

**Affiliations:** a The University of Arizona, James C. Wyant College of Optical Sciences, Tucson, Arizona, United States; b The University of Arizona, College of Medicine, Department of Medicine, Division of Dermatology, Tucson, Arizona, United States

**Keywords:** dermatoscopy, dermoscopy, smartphone imaging, multispectral imaging, mHealth

## Abstract

**Significance:** The rates of melanoma and nonmelanoma skin cancer are rising across the globe. Due to a shortage of board-certified dermatologists, the burden of dermal lesion screening and erythema monitoring has fallen to primary care physicians (PCPs). An adjunctive device for lesion screening and erythema monitoring would be beneficial because PCPs are not typically extensively trained in dermatological care.

**Aim:** We aim to examine the feasibility of using a smartphone-camera-based dermascope and a USB-camera-based dermascope utilizing polarized white-light imaging (PWLI) and polarized multispectral imaging (PMSI) to map dermal chromophores and erythema.

**Approach:** Two dermascopes integrating LED-based PWLI and PMSI with both a smartphone-based camera and a USB-connected camera were developed to capture images of dermal lesions and erythema. Image processing algorithms were implemented to provide chromophore concentrations and redness measures.

**Results:** PWLI images were successfully converted to an alternate colorspace for erythema measures, and the spectral bandwidth of the PMSI LED illumination was sufficient for mapping of deoxyhemoglobin, oxyhemoglobin, and melanin chromophores. Both types of dermascopes were able to achieve similar relative concentration results.

**Conclusion:** Chromophore mapping and erythema monitoring are feasible with PWLI and PMSI using LED illumination and smartphone-based cameras. These systems can provide a simpler, more portable geometry and reduce device costs compared with interference-filter-based or spectrometer-based clinical-grade systems. Future research should include a rigorous clinical trial to collect longitudinal data and a large enough dataset to train and implement a machine learning-based image classifier.

## 1 Introduction

The rates of melanoma and nonmelanoma skin cancers (NMSC) have been steadily rising,^1^^,^^2^ and early diagnosis is key for improved outcomes.^3^ Because there is a shortage of board-certified dermatologists,^4^^,^^5^ particularly in remote or underserved settings where <10% of dermatologists practice,^6^ most of the burden of diagnosis and treatment falls on primary care physicians (PCPs) who are not extensively trained in dermatological care.^3^^,^^7^ Dermoscopy is a tool utilized to improve the *in vivo* diagnostic accuracy of benign versus malignant lesions, a unique skill that requires additional training, even among board-certified dermatologists. In remote settings, dermascopes may capture and document pigmented lesions that can be forwarded to expert colleagues through telemedicine for further analysis.^8^ Unfortunately, dermascopes and their accessories range from hundreds to thousands of dollars,^9^^,^^10^ which is potentially too expensive for general medical practice. Thus, there is a need for a low-cost, readily available dermoscopy tool to bridge this clinical need.

Lesion evaluation using visual, subjective methods such as the ABCDE criteria and seven-point checklist are useful tools for PCPs.^3^^,^^11^ The ABCDE criteria predict melanoma by a lesion’s asymmetry, border irregularity, coloration, diameter if >6  mm, and evolution, providing a sensitivity of 0.85 and specificity of 0.72.^3^^,^^11^ The seven-point checklist monitors a lesion’s change in size, shape, color, and looks for diameters >7  mm, crusting or bleeding, and sensory change, providing a sensitivity of 0.77 and specificity of 0.80.^3^ Continuous monitoring has shown to improve outcomes through early detection as evidenced by mole mapping techniques^12^^,^^13^ and the increase in sensitivity and specificity with the addition of the evolving in the ABCDE criteria.^11^

Adjunctive tools utilizing objective measures such as polarized multispectral imaging (PMSI) and polarized white-light imaging (PWLI) to map dermal chromophores [hemoglobin, deoxyhemoglobin (Hb), and melanin], quantify erythema, and perform image classification for lesion screening have the potential to increase early detection of melanoma by PCPs and even outside the physician’s office, leading to reduced need for biopsy and improved outcomes.^14^^–^^27^ We propose a smartphone combined with LED illumination as the ideal platform for an adjunctive medical device, which will provide a portable system with easy-to-operate apps and native image capture, processing, and data transmission. These systems can reduce the costs associated with interference-filter-based^14^^,^^15^^,^^20^ or spectrometer-based^21^^,^^23^ systems while also providing a more compact, portable geometry for use in any testing environment compared with clinical-grade imaging systems.^17^^–^^19^

## 2 Materials

We have developed two point-of-care dermascope design concepts for skin lesion screening and erythema monitoring, implementing both PMSI and PWLI^28^ on an LG G5 (LG, Seoul, South Korea) smartphone platform. One system concept utilizes the embedded smartphone camera for imaging while the other uses a USB-connected camera module that connects to the smartphone. Both systems share a common illumination system and software application to enable PWLI and PMSI.

**Fig. 1:**
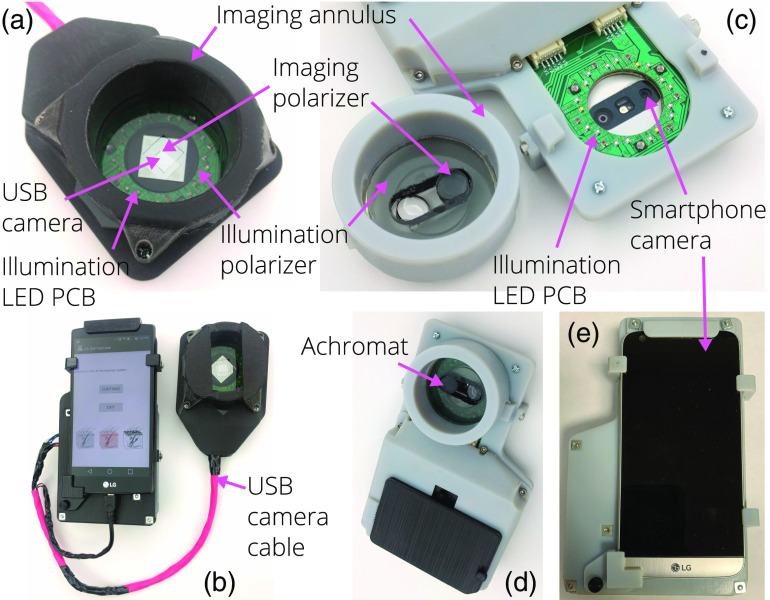
Two dermascope implementations. The USB-camera-based PMSI and PWLI dermascope is shown in (a) and (b). (a) Various components of the handheld imaging module (the USB camera is hidden behind the imaging polarizer) and (b) the imaging module paired with the smartphone camera. The smartphone-camera-based PMSI and PWLI dermascope is shown in (c), (d), and (e). (c) The smartphone-based system’s side opposite the smartphone screen with the imaging annulus removed, where the LED PCB and smartphone camera are visible and other components are highlighted; (d) the system with the imaging annulus attached; and (e) the smartphone installed in the dermascope.

The PMSI and PWLI dermascope using the smartphone’s embedded rear camera is shown in Figs. 1(c)–1(e). The main LG G5 camera consists of a Sony IMX234 Exmor RS sensor with 5312×2988, 1.12-μm pixels and a 5.95  mm×3.35  mm sensor size. The sensor is paired with a f/1.8, 4.42-mm focal length lens.

**Fig. 2:**
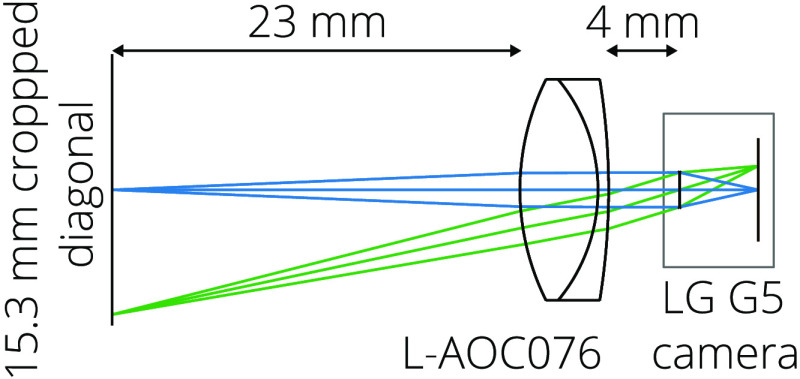
Layout of the LG G5 smartphone camera and the added achromat. The smartphone camera lens system is modeled as a paraxial lens.

To decrease the working distance of the optical system to allow imaging of the epidermis, a 24-mm focal length achromatic doublet (Ross Optical, El Paso, Texas, USA) is placed 4 mm away from the principal plane of the smartphone optical system, providing a magnification of m=0.187 and a numerical aperture NA=0.04. After cropping, the field of view (FOV) is 9.96  mm×11.67  mm, as shown in Fig. 2. The imaging achromat is aligned to the smartphone camera using a machined PMMA disk installed in a removable 3D-printed annulus of VeroBlue RGD840 (Stratasys, Eden Prairie, Minnesota, USA) plastic. The annulus serves as an imaging guide; its length equals the optical system working distance (23 mm), so the PCP can contact the patient to stabilize the device and ensure correct focus. An additional 3D-printed structure serves as a mounting platform for the smartphone, imaging annulus, and LED electronics.

The alternative PMSI and PWLI dermascope [Figs. 1(a) and 1(b)] is also based on an LG smartphone platform, but it utilizes an external USB-connected RGB camera (OV5648, Omnivision, Santa Clara, California, USA; 5 MP, 3.67  mm×2.74  mm) with the vendor-supplied ∼2.8-mm focal length lens adjusted to a working distance of 30 mm. After cropping, the FOV is 27.5  mm×20  mm. In addition, the integrated infrared (IR) filter was removed. Again, the mechanical design of the annulus is matched to the working distance of the camera, providing in-focus imaging when the device contacts the patient.

**Fig. 3:**
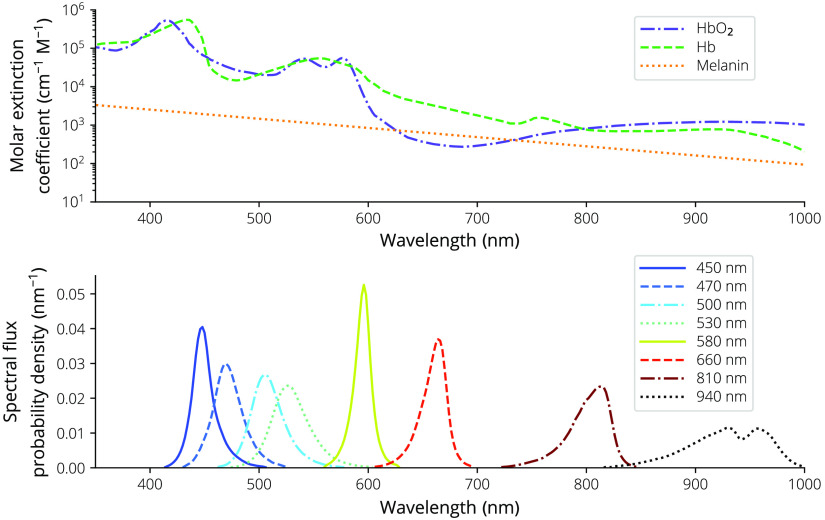
Molar extinction coefficients, ε(λ), for Hb, ${\mathrm{HbO}}_{2}$, and melanin plotted on a log scale and the LED spectral flux probability density functions, $\phi_{e,\lambda }$, plotted on a linear scale.

For both systems, multispectral illumination is accomplished using a custom printed circuit board (PCB) with LEDs of various wavelengths (Lumileds, Amsterdam, The Netherlands; Vishay, Malvern, Pennsylvania, USA) installed as shown in Table 1. The color wavelengths were chosen based on commercial availability and the ability to probe both hemoglobin isosbestic points and separate oxygenated from deoxygenated hemoglobin content along the molar attenuation curves (Fig. 3).

**Table 1:** LED and camera settings for each illumination wavelength and each dermascope. LED wavelength (λ), LED part number, smartphone camera LED-driving current (I), smartphone camera LED flux for a single LED of the given color, smartphone camera International Organization for Standardization setting, smartphone camera exposure time, USB camera LED-driving current (I), USB camera LED flux for a single LED of the given color, USB camera brightness setting, and USB exposure time are provided.

λ (nm)	Part number	Smartphone camera settings				USB camera settings			
		I (mA)	Flux	ISO	Exposure time (ms)	I (mA)	Flux	Brightness	Exposure time (s)
4000 K	LXZ1-4070	358	101 lm	100	3.8	620	161 lm	50	1.6
450	LXZ1-PR01	620	690 mW	100	0.5	620	690 mW	50	1.6
470	LXZ1-PB01	620	46 lm	100	0.7	620	46 lm	50	1.6
500	LXZ1-PE01	620	100 lm	100	2.6	620	100 lm	50	1.6
530	LXZ1-PM01	620	142 lm	100	3.0	620	142 lm	50	1.6
580	LXZ1-PL01	358	42 lm	100	5.0	620	67 lm	50	1.6
660	LXZ1-PA01	620	420 mW	100	0.9	620	420 mW	50	1.6
810	VSMY98145DS	620	700 mW	2390	250.0	620	700 mW	50	1.6
940	L1IZ-0940	358	403 mW	2300	180.0	620	700 mW	50	1.6

For the smartphone-based dermascope, the PMMA disk used for mounting the lens also extends over the illumination LEDs to provide mounting for a linear polarizer (Edmund Optics, Barrington, New Jersey, USA). An orthogonal linear polarizer is installed in front of the imaging channel, enabling both PMSI and PWLI and reducing the effect of specular reflection on the images.^28^ The LED sources’ spectral fluxes, $\phi_{e,\lambda }$, shown in Fig. 3, were measured with a spectrometer (Ocean Optics).

The USB-camera-based dermascope uses the same LED PCB and wavelengths for illumination along with orthogonal polarizers in the illumination channel (Edmund Optics) and the imaging channel (Moxtek, Orem, Utah, USA). To help normalize white-light image luminance, an 18% gray color reference (Kodak, Rochester, New York, USA) is permanently installed on both sides of the image FOV. Because the 3D-printed mounting foundation does not need to mount the LED board and imaging annulus, a previously designed geometry is used for this system.^29^

**Fig. 4:**
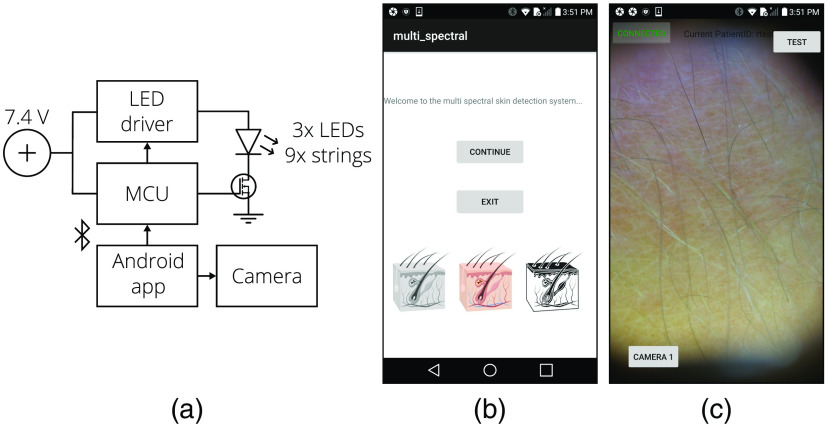
The system electronics block diagram is provided in (a) and Android application screenshots in (b) and (c).

The illumination PCB consists of three LEDs of each color soldered in a symmetrical pattern around the camera aperture to maximize uniformity without additional beam shaping optics. The backside solder mask of the PCB was removed to expose the copper and is attached to a copper heatsink with electrically insulating epoxy (DP240, 3M, St. Paul, Minnesota, USA). Numerous vias were placed on the PCB to ensure a low thermal resistance between the front and backside copper planes. The LEDs are driven with a switching boost power supply (LT3478, Linear Technology, Milpitas, California, USA) powered by two lithium-ion batteries (Orbtronic, Saint Petersburg, Florida, USA). Each LED color string can be turned on individually with a custom power level setting and illumination, and image capture is synchronized by a custom Android application through a Bluetooth-connected microcontroller (MCU, IOIO-OTG, SparkFun Electronics, Niwot, Colorado, USA). The LED-driving currents, fluxes, and dermascopes’ image capture settings are shown in Table 1. In addition, the smartphone camera uses the daylight white balance setting, and the white balance setting of the USB camera is inaccessible. A block diagram of the system electronics is shown in Fig. 4.^30^ The Android application controls the camera functions, synchronizes the LED illumination, and sets camera exposure time. For the USB camera, the Android app was modified to use the USB camera instead of the on-board smartphone camera. Images are connected to an ID assigned to each patient, removing identifiable information from the smartphone. Screenshots of the app are shown in Fig. 4.

## 3 Methods

### 3.1 Data Processing

The algorithms used to process collected dermal images are provided in Algorithms 1 and 2. Descriptions of the steps and related equations are provided in the following sections.

**Algorithm 1:** Processing of reference images.

1: **procedure** ProcessReferenceImages (reference images)
2: **for all** reference images **do**
3: convert sRGB to linear RGB ▹ Eq. (1)
4: **if** white-light image **then**
5: convert linear RGB to CIEXYZ ▹ Eq. (2)
6: **else if** color image **then**
7: convert linear RGB to Yequal ▹ Eq. (3)
8: **end if**
9: calculate luminance reference ▹ Eq. (4)
10: calculate illumination uniformity reference ▹ Eq. (5)
11: **end for**
12: **return** optical density reference images
13: **return** illumination uniformity images
14: **end procedure**

**Algorithm 2:** Processing of dermal images.

1: **procedure** ProcessDermalImages (dermal images)
2: **for all** dermal images **do**
3: **if** USB camera **then**
4: correct white-light image luminance ▹ Eq. (8)
5: **end if**
6: convert sRGB to linear RGB ▹ Eq. (1)
7: correct by illumination uniformity ▹ Eq. (6)
8: **if** white-light image **then**
9: convert linearRGB to CIEXYZ ▹ Eq. (2)
10: convert CIEXYZ to CIELAB ▹ Eqs. (17) and (18)
11: **else if** color image **then**
12: convert linear RGB to Yequal ▹ Eq. (3)
13: calculate optical density ▹ Eq. (7)
14: calculate melanin content ▹ Eq. (19)
15: calculate erythema ▹ Eq. (20)
16: solve chromophore concentration ▹ Eq. (14)
17: **end if**
18: **end for**
19: **end procedure**

#### 3.1.1 Image collection

When the dermascopes were first built, images of an 18% reflective gray card were collected by each system at each wavelength to serve as both the optical density (OD) and illumination uniformity references.

For dermal image collection, a pilot study was performed on human subjects at the University of Arizona College of Medicine, Division of Dermatology to determine feasibility of each multispectral dermascope. This study received institutional review board approval (#1612067061). All patients provided informed written and oral consent.

#### 3.1.2 Colorspace conversions

The melanin content, erythema, and chromophore concentration measurements rely on conversion to the CIELAB and CIEXYZ colorspaces. The imaging systems natively capture in the sRGB colorspace, and the images are first converted to linear RGB space:^31^

$$C_{\text{linear}}=\{\begin{array}{ll} \frac{C_{\mathrm{sRGB}}}{12.92} & C_{\mathrm{sRGB}}\leq 0.04045\\ {\left(\frac{C_{\mathrm{sRGB}}+0.055}{1+0.055}\right)}^{2.4} & C_{\mathrm{sRGB}}>0.04045 \end{array},$$ (1)

where $C_{\mathrm{sRGB}}$ is each channel of the $I_{\mathrm{sRGB}}$ image. Images are then converted from ${\mathrm{RGB}}_{\text{linear}}$ to CIEXYZ using the transformation matrix,^31^

$$[\begin{array}{c} X\\ Y\\ Z \end{array}]=[\begin{array}{ccc} 0.4124 & 0.3576 & 0.1805\\ 0.2126 & 0.7152 & 0.0722\\ 0.0193 & 0.1192 & 0.9505 \end{array}]\cdot [\begin{array}{c} R_{\text{linear}}\\ G_{\text{linear}}\\ B_{\text{linear}} \end{array}],$$ (2)

where Y is the luminance value and is used to calculate ODs from the dermis images and reference. Luminance is a measure that scales optical radiation by the response of the human visual system.^32^ Because the images will be processed by a computer, accurate color representation for a human is not required, so an additional luminance measure, $Y_{\text{equal}}$, is created using the equal sum of all three channels:

$$Y_{\text{equal}}=[\begin{array}{ccc} 1 & 1 & 1 \end{array}]\cdot [\begin{array}{c} R_{\text{linear}}\\ G_{\text{linear}}\\ B_{\text{linear}} \end{array}].$$ (3)

#### 3.1.3 Reference and illumination uniformity correction

Using the reference images that have been converted to CIEXYZ or Yequal, reference luminance images are defined as

$$I_{0}=\overset{¯}{Y_{\mathrm{ref}}},$$ (4)

where $Y_{\mathrm{ref}}$ is the Y (luminance) channel of the CIEXYZ image or Yequal. The reference grayscale image is normalized to serve as the illumination reference for the dermal images.

$$U=\frac{Y_{\mathrm{ref}}}{\max(Y_{\mathrm{ref}})},$$ (5)

where U is now the illumination uniformity correction matrix.

The dermal CIEXYZ and Yequal images are corrected in the same way

$$I_{\text{dermal},\text{uniformity corrected}}=\frac{I_{\text{dermal}}}{U}\overset{¯}{U},$$ (6)

where $I_{\text{dermal}}$ is the illumination uniformity corrected dermal image with constant mean luminance. Finally, OD dermal images are calculated as

$$\mathrm{OD}=-\ln(\frac{I}{I_{0}}).$$ (7)

Finally, the USB dermascope has sections of a 18% gray photography card mounted on either side of the FOV [Fig. 1(b)]. Knowing the card image should equal 50% levels of RGB, the luminance of the white-light image is scaled using the following equation:

$$I_{\text{dermal},\text{luminance corrected}}=I_{\text{dermal},\text{uniformity corrected}}\frac{0.5}{\overset{¯}{Y_{\text{gray}}}}.$$ (8)

#### 3.1.4 Chromophore concentration

The Beer–Lambert law is utilized to measure the relative concentrations of Hb, oxyhemoglobin (${\mathrm{HbO}}_{2}$), and melanin:^17^^,^^22^^,^^33^^–^^35^

$$I(\lambda )=I_{0}(\lambda )\exp[-c_{n}\varepsilon (\lambda )\ell (\lambda )],$$ (9)

where I is the resulting intensity, $I_{0}$ is the incident intensity, $c_{n}$ is the concentration of the chromophore, ε(λ) is the molar attenuation coefficient of the chromophore at a particular wavelength, and ℓ(λ) is the optical path length of the light in the medium for the incident wavelength. This is restated as OD:

$$\mathrm{OD}=-\log(\frac{I(\lambda )}{I_{0}(\lambda )})=c_{\mathrm{Hb}}\varepsilon_{\mathrm{Hb}}(\lambda )\ell (\lambda )+c_{{\mathrm{HbO}}_{2}}\varepsilon_{{\mathrm{HbO}}_{2}}(\lambda )\ell (\lambda )\;+c_{\text{melanin}}\varepsilon_{\text{melanin}}(\lambda )\ell (\lambda )+c_{\text{background}},$$ (10)

where $c_{\text{background}}$ is due to residual absorption from molecules present in the epidermis and dermis.

The molar extinction coefficients for Hb and ${\mathrm{HbO}}_{2}$^36^ and melanin^37^ are shown in Fig. 3. Jacques’s $\varepsilon_{\text{melanin}}$^37^ was fit with an exponential curve to extend the wavelength to 1000 nm, resulting in a fit of

$$\varepsilon_{\text{melanin}}=2.2858\cdot 10^{4}\exp(-5.5028\cdot 10^{-3}\lambda ).$$ (11)

Optical path lengths, ℓ(λ), for the chromophores are calculated from a linear fit of Anderson’s data^38^ in the region of the illumination wavelengths,

$$\ell (\lambda )=2.62\cdot 10^{-4}\lambda -9.87\cdot 10^{-2},$$ (12)

where λ is in units of nm and ℓ(λ) is in units of cm.

Because the LEDs are broad spectrum, we integrate over the wavelength probability density function to calculate a total molar attenuation coefficient^39^^,^^40^ for each color

$$\varepsilon_{\text{total}}=\int \phi_{e,\lambda }(\lambda )\varepsilon (\lambda )d\lambda .$$ (13)

The resulting molar attenuation coefficients for all of the chromophores are shown in Table 2.

**Table 2:** Molar extinction coefficients calculated using Eq. (13) for each illumination wavelength compared with the molar extinction coefficients for the peak wavelength.

Wavelength (nm)	Coefficients from Eq. (13)			Coefficients at peak LED wavelength		
	Hb (${\mathrm{cm}}^{-1}\;M^{-1}$)	${\mathrm{HbO}}_{2}$ (${\mathrm{cm}}^{-1}\;M^{-1}$)	Melanin (${\mathrm{cm}}^{-1}\;M^{-1}$)	Hb (${\mathrm{cm}}^{-1}\;M^{-1}$)	${\mathrm{HbO}}_{2}$ (${\mathrm{cm}}^{-1}\;M^{-1}$)	Melanin (${\mathrm{cm}}^{-1}\;M^{-1}$)
450	199,864	82,747	1922	103,292	62,816	1921
470	35,937	36,662	1706	16,156	33,209	1721
500	26,659	25,521	1392	20,862	20,932	1459
530	37,824	34,851	1241	39,036	39,957	1237
580	22,606	13,258	869	37,010	50,104	940
660	3380	352	611	3227	320	605
810	845	812	281	717	864	265
940	656	1185	142	693	1214	130

A system of equations is built from the multispectral datacube and the molar attenuation coefficients shown in Table 2

$$[\begin{array}{cccc} \varepsilon_{\mathrm{Hb}}(\lambda_{1})\ell (\lambda_{1}) & \varepsilon_{{\mathrm{HbO}}_{2}}(\lambda_{1})\ell (\lambda_{1}) & \varepsilon_{\text{melanin}}(\lambda_{1})\ell (\lambda_{1}) & 1\\ \varepsilon_{\mathrm{Hb}}(\lambda_{2})\ell (\lambda_{2}) & \varepsilon_{{\mathrm{HbO}}_{2}}(\lambda_{2})\ell (\lambda_{2}) & \varepsilon_{\text{melanin}}(\lambda_{2})\ell (\lambda_{2}) & 1\\ \varepsilon_{\mathrm{Hb}}(\lambda_{3})\ell (\lambda_{3}) & \varepsilon_{{\mathrm{HbO}}_{2}}(\lambda_{3})\ell (\lambda_{3}) & \varepsilon_{\text{melanin}}(\lambda_{3})\ell (\lambda_{3}) & 1\\ \vdots & \vdots & \vdots & \vdots \\ \varepsilon_{\mathrm{Hb}}(\lambda_{n})\ell (\lambda_{n}) & \varepsilon_{{\mathrm{HbO}}_{2}}(\lambda_{n})\ell (\lambda_{n}) & \varepsilon_{\text{melanin}}(\lambda_{n})\ell (\lambda_{n}) & 1 \end{array}][\begin{array}{c} c_{\mathrm{Hb}}\\ c_{{\mathrm{HbO}}_{2}}\\ c_{\text{melanin}}\\ c_{\text{background}} \end{array}]=[\begin{array}{c} \mathrm{OD}(\lambda_{1})\\ \mathrm{OD}(\lambda_{2})\\ \mathrm{OD}(\lambda_{3})\\ \vdots \\ \mathrm{OD}(\lambda_{n}) \end{array}].$$ (14)

and the system is solved by linear algebra least-squares techniques^33^ where $\mathrm{OD}(\lambda_{n})$ are calculated OD matrices for each illumination wavelength.

The ability of the dermascopes to properly measure relative chromophore concentrations was validated using a finger occlusion test. Images were taken with both dermascopes and the chromophores mapped preocclusion, after 2 min of occlusion, postocclusion, and 5 min after ending the occlusion.^41^

#### 3.1.5 Melanin and erythema

**Fig. 5:**
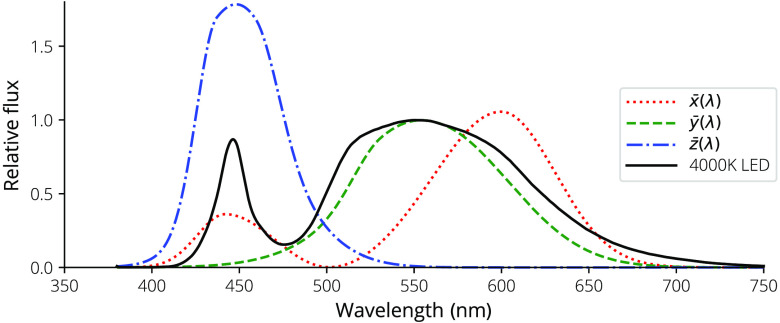
Color matching curves used to determine normalization constants to convert to CIELAB along with the 4000 K white-LED spectrum.

To measure melanin content and erythema, the white-light image is converted to the CIELAB^42^ colorspace using lightness ($L^{*}$) as a measure of relative melanin content and the direction of red color stimuli ($a^{*}$) as a measure of redness, with more positive values indicating higher levels of erythema.^43^ Before converting to CIELAB, normalization constants must be calculated from the white-LED spectral content. Using the color matching functions,^44^ $\overset{¯}{x}(\lambda )$, $\bar{y}(\lambda )$, $\overset{¯}{z}(\lambda )$ (Fig. 5), X, Y, and Z are calculated as^42^

$$X=\int_{380\;\mathrm{nm}}^{780\;\mathrm{nm}}\overset{¯}{x}(\lambda )\phi_{e,\lambda }d\lambda ;\;Y=\int_{380\;\mathrm{nm}}^{780\;\mathrm{nm}}\overset{¯}{y}(\lambda )\phi_{e,\lambda }d\lambda ;\;Z=\int_{380\;\mathrm{nm}}^{780\;\mathrm{nm}}\overset{¯}{z}(\lambda )\phi_{e,\lambda }d\lambda ,$$ (15)

where $\phi_{e,\lambda }$ is the relative spectral flux of the white-LED source as shown in Fig 3. The normalization constants $X_{n}$, $Y_{n}$, and $Z_{n}$ are calculated by

$$X_{n}=\frac{X}{Y};\;Y_{n}=\frac{Y}{Y};\;Z_{n}=\frac{Z}{Y}.$$ (16)

The image is then converted to CIELAB by^42^

$$L^{*}=116f(\frac{Y}{Y_{n}})-16,\;a^{*}=500[f(\frac{X}{X_{n}})-f(\frac{Y}{Y_{n}})],\;b^{*}=200[f(\frac{Y}{Y_{n}})-f(\frac{Z}{Z_{n}})],$$ (17)

where

$$f(x)=\{\begin{array}{ll} x^{1/3} & x>{\left(24/116\right)}^{3}\\ (841/108)x+16/116 & x\leq {\left(24/116\right)}^{3} \end{array}.$$ (18)

In addition to the white-light image measures, melanin and erythema measures are constructed from the color-OD images. Melanin content^16^^,^^45^ is calculated as

$$\text{Melanin}={\mathrm{OD}}_{660}-{\mathrm{OD}}_{940}.$$ (19)

As shown in Table 2, these two wavelengths maximize the difference in melanin absorption and minimize the effect of Hb and ${\mathrm{HbO}}_{2}$ absorption.

Erythema, due to increased blood content, results in increased blue light absorption but little change in red light absorption^46^ as shown in Table 2. Therefore, an erythema index is constructed as

$$\text{Erythema}={\mathrm{OD}}_{470}-{\mathrm{OD}}_{660}.$$ (20)

### 3.2 Optical System Characterization

The linearity of the camera responses was measured by adjusting the exposure time in the case of the smartphone-camera-based dermascope and image brightness in the case of the USB-camera-based dermascope, capturing images of the matte 18% gray photography card with each LED color, and measuring the image luminance mean at each wavelength.

Performance of the imaging system’s cutoff frequency and FOV was validated with a 1951 United States Air Force (USAF) resolution test chart, and the modulation transfer function (MTF) was measured using the slanted-edge method.^47^

Illumination uniformity was measured by illuminating the matte 18% gray photography card with each LED color and imaging the surface with the dermascope. The uniformity is quantified using the coefficient of variation, ($c_{v}$),^48^ on normalized data

$$\text{Uniformity}=1-c_{v}=1-\frac{\sigma }{\overset{¯}{x}},$$ (21)

where $\bar{x}$ is the mean of the pixels in the image and σ is the standard deviation of the pixel values.

## 4 Results

### 4.1 Clinical Results

**Fig. 6:**
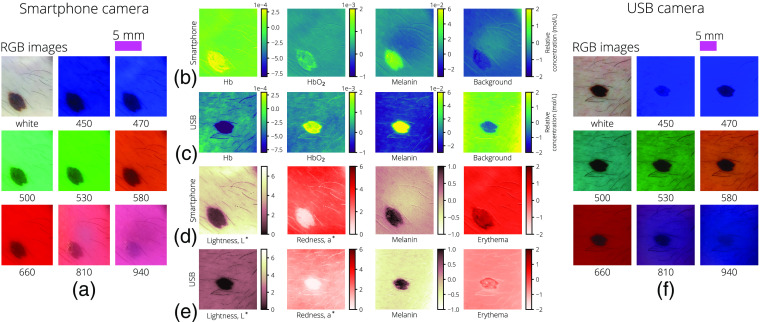
The same JN imaged by both the smartphone and USB dermascopes. For the smartphone dermascope, (a) the RGB images after illumination uniformity correction, (b) the relative chromophore concentrations, and (d) lightness as measured by $L^{*}$, redness as measured by $a^{*}$, melanin calculated from Eq. (19), and erythema calculated from Eq. (20). The same measures are shown for the USB dermascope in (f), (c), and (e), respectively. A 5-mm scale bar is provided for both the smartphone-camera images and the USB camera images above the RGB image grids.

**Fig. 7:**
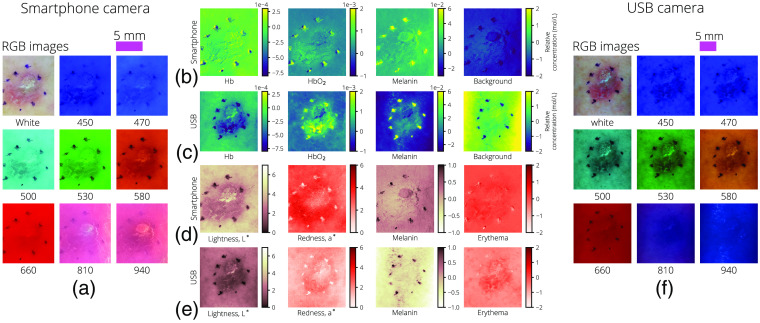
The same SCC imaged by both the smartphone and USB dermascopes. For the smartphone dermascope, (a) the RGB images after illumination uniformity correction, (b) the relative chromophore concentrations, and (d) lightness as measured by $L^{*}$, redness as measured by $a^{*}$, melanin calculated from Eq. (19), and erythema calculated from Eq. (20). The same measures are shown for the USB dermascope in (f), (c), and (e), respectively. A 5-mm scale bar is provided for both the smartphone-camera images and the USB camera images above the RGB image grids.

Following are the RGB, chromophore, melanin, and erythema measures for cases of junctional nevus (JN) (Fig. 6) and squamous cell carcinoma (SCC) (Fig. 7); each case was captured with both the USB camera dermascope and the smartphone camera dermascope.

**Fig. 8:**
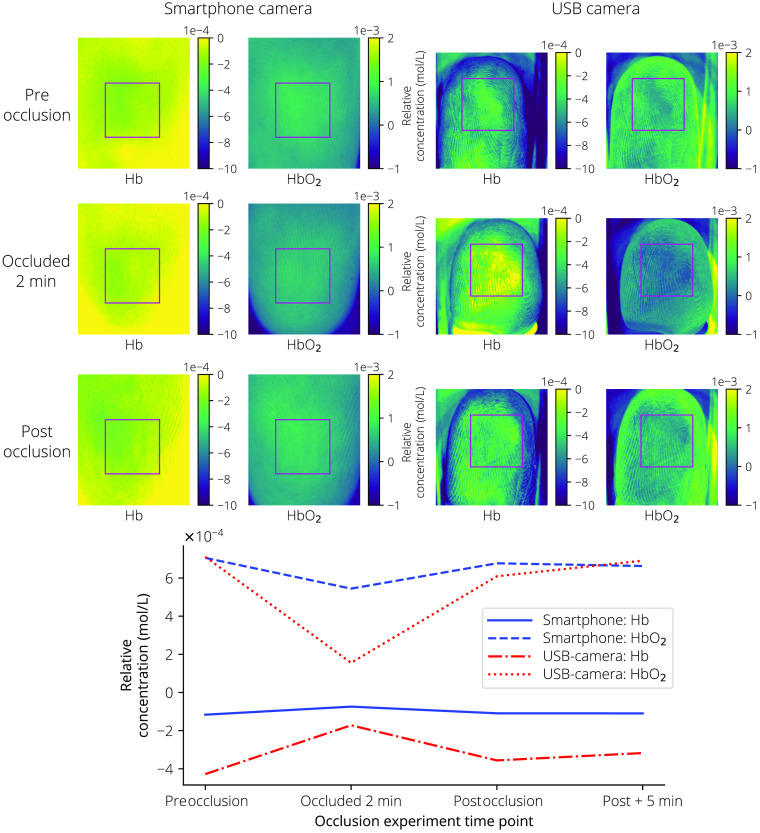
Finger occlusion test results for the smartphone camera and USB camera at preocclusion, after occlusion for 2 min, and postocclusion. The bottom plot provides the mean relative concentration for Hb and ${\mathrm{HbO}}_{2}$ inside the rectangle showing a dip in ${\mathrm{HbO}}_{2}$ and increase in Hb after occlusion.

The chromophore maps for both dermascopes at the chosen time points for the occlusion test are shown in Fig. 8.

### 4.2 Optical System Performance

**Fig. 9:**
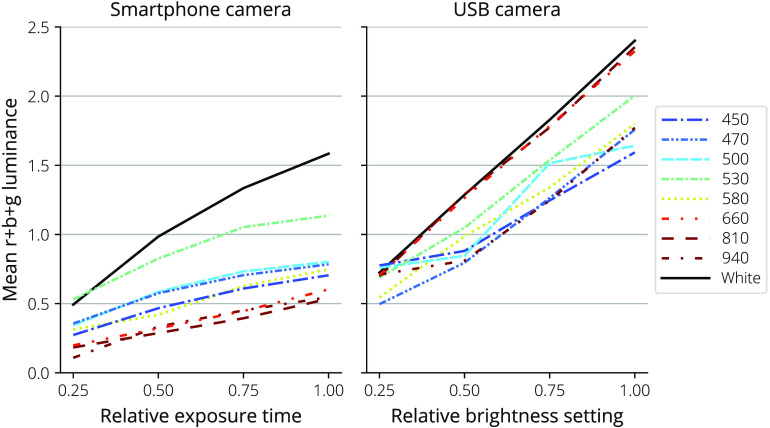
Mean of the sum of the red, green, and blue channels over changing exposure times for the smartphone-based camera and changing brightness settings for the USB camera.

Figure 9 shows the changes in the mean of the sum of the red, green, and blue image channels over varying exposure times for the smartphone-based camera and over brightness settings for the USB camera.

**Fig. 10:**
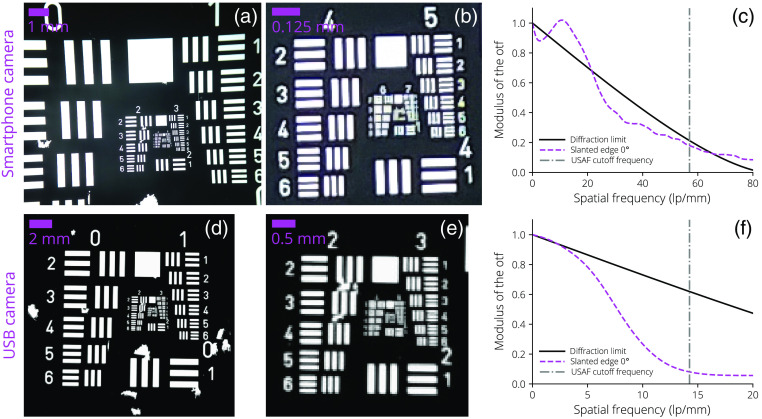
(a)–(c) Smartphone camera and (d)–(f) USB camera results of 1951 USAF resolution test chart imaging along with measured MTFs from a slanted-edge test. The smartphone camera’s measured MTF matches the USAF cutoff frequency of group 5 to 6 (57  lp/mm). The USB camera’s measured MTF matches the USAF cutoff frequency of group 3 to 6 (14.25  lp/mm).

Figure 10 shows full-field and zoomed 1951 USAF resolution test chart images after cropping along with measured MTF data using the slanted-edge test for both dermascopes.

**Fig. 11:**
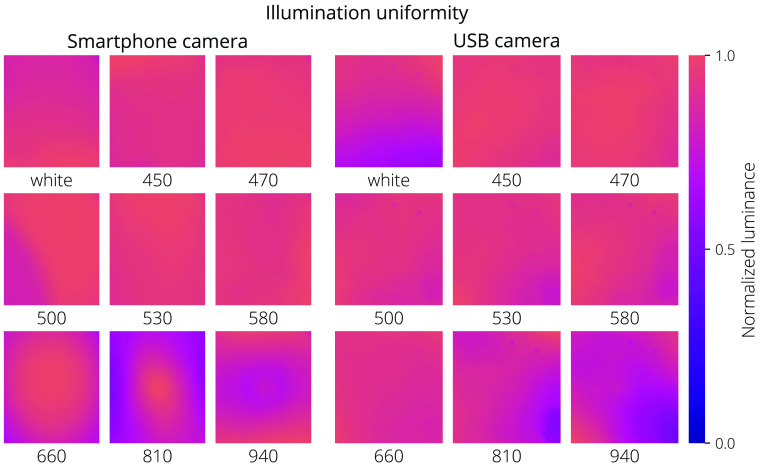
Normalized luminance maps showing illumination uniformity of each device and each illumination wavelength corresponding to U in Eq. (5)

Maps of the illumination uniformities of both systems are shown in Fig. 11, and the coefficient of variations are given in Table 3.

**Table 3:** LED illumination uniformity according to Eq. (21).

Wavelength	Smartphone camera	USB camera
White	0.954	0.880
450	0.974	0.980
470	0.982	0.977
500	0.935	0.969
530	0.977	0.955
580	0.980	0.949
660	0.916	0.972
810	0.852	0.900
940	0.907	0.870

### 4.3 CIEXYZ Normalization

The CIEXYZ normalization constants calculated from the white-LED spectrum for the two dermascopes are shown in Table 4.

**Table 4:** Measured CIEXYZ normalization constants for both dermascopes.

	Smartphone camera	USB camera
$X_{n}$	82.873	82.846
$Y_{n}$	100	100
$Z_{n}$	34.567	48.757

## 5 Discussion

The distribution of polarized multispectral dermascopes based on smartphone platforms and low-cost color LEDs to PCPs (and eventually to consumers) has the potential to democratize dermal chromophore and melanoma mapping along with erythema monitoring, improving quantitative monitoring of lesions and increasing early detection of skin cancers.

This platform demonstrates a number of advantages compared with previous systems targeting chromophore mapping and skin cancer screening.^14^^,^^15^^,^^17^^,^^18^^,^^20^^,^^22^^–^^24^ The smartphone platform is a compact, low-cost, portable, easy-to-use system with native image capture and processing capabilities, which removes the need for expensive, clinical-grade imaging systems.^17^^–^^19^ The platform is flexible enough to use either the embedded camera for imaging or a separate USB-connected camera, depending on the desired ergonomics of the user. Both system implementations can still use the built-in smartphone camera for wide-field, white-light, and dermal imaging [the annulus in Fig. 1(c) can be removed]. Additionally, the smartphone camera can be used for large area image capture either using the smartphone-camera-based dermascope with the imaging annulus removed or using the USB camera’s host smartphone.

The use of low-cost, compact, high-power, high-efficacy, surface mount LEDs improves on the costs and complexities associated with laser-based,^22^^,^^24^ interference-filter-based,^14^^,^^15^^,^^20^ and spectrometer-based^21^^,^^23^ systems. While these systems likely allow for better discrimination due to their narrow-bandwidth sources or detection schemes, the costs involved (with the possible exception of the laser-based systems) are prohibitive. High-reliability LEDs are available in myriad wavelengths to probe various points along the chromophore molar attenuation curves (Fig. 3) and can be powered with simple driving circuits. Surface-mount packages remove the bulk of transistor outline can packages (or larger packages) necessary for edge-emitting lasers, and the broad wavelength selection is wider than that of surface mount laser packages such as vertical-cavity surface-emitting lasers. The cost of LED sources compared with laser sources or interference filters allows for the use of multiple wavelengths in a single system while keeping bill of materials (BOM) costs low.

### 5.1 Clinical Testing

Initial testing of the systems is promising as both systems were able to capture full image datasets and return similar results of relative chromophore concentrations across multiple dermal lesions except for Hb in the JN case, as shown in Fig. 6. The deviation could be explained by the difference in IR imaging performance between the two dermascopes.

In addition, relative melanin content and erythema as measured through the CIELAB white-light images and OD color images agreed between systems and are reasonable based on visual examination. The USB camera and smartphone camera have differing levels of luminance in their white-light images as seen in Figs. 6 and 7, leading to a difference in baseline lightness and redness values, where the higher luminance smartphone images show higher overall $L^{*}$ and $a^{*}$ values. However, as seen in Fig. 6, the relative changes are similar, where $\Delta L^{*}\approx 3$ between the nevus and surrounding skin and $\Delta a^{*}\approx 3$ between the nevus and surrounding skin.

The occlusion test (Fig. 8) provided directionally correct results for both dermascopes, although the magnitudes of change in chromophore concentration were dissimilar between dermascopes. Again, this deviation could be explained by the difference in IR imaging performance between the two dermascopes. With the next system revision, the ability to measure absolute concentrations should be confirmed with known blood and melanin phantoms.

To fully validate the system, a full clinical trial of longitudinal data with multiple types of skin lesions in addition to testing patients with a wide range of baseline melanin levels will be necessary.^49^ Once a large dataset is collected along with biopsy and diagnosis results, classification algorithms can be built using machine learning, principal components analysis, or similar tools.^25^^–^^27^^,^^50^ The statistics of the large dataset and the classifier can then be used to predict the relationships between chromophores, lesion type, and diagnosis. In our two datasets, high-melanin concentrations were present for the JN case but not for the SCC case. The classifier will help to determine if this relationship is true more generally or how this might change in patients with high baseline levels of melanin. Likewise, while the Hb and ${\mathrm{HbO}}_{2}$ levels were similar in our two datasets, a larger dataset might reveal that cancerous activity increases blood flow,^51^ increasing both Hb and ${\mathrm{HbO}}_{2}$ and possibly the ratios between them. The classifier could use additional features and relationships in the images. For example, by Eq. (12), the optical path length increases as the wavelength increases, increasing the probe depth. Detecting lesion shape changes over depth through edge detection or similar means could provide another layer of information. Hints of these changes are apparent in both the JN and SCC cases as both have changing edges as the wavelength changes. Likewise, the classifier could potentially use additional measures such as blood contrasts^16^ and oxygenation percentages.^52^

### 5.2 Measured Optical Performance

Both cameras produced approximately linear responses when changing exposure time in the case of the smartphone camera dermascope and brightness in the case of the USB camera, providing confidence in the ability of the systems to have a linear response to intensity changes from illumination absorption.

For the smartphone dermascope, the measured MTF performance matched both the predicted diffraction-limited performance and the cutoff frequency measured with the USAF target where group 5 to 6 (57  lp/mm) is resolvable. The root mean square error (RMSE) between the measured MTF and predicted diffraction-limited performance was RMSE=0.97. The USB dermascope’s measured MTF performance did not match the predicted diffraction-limited performance (RMSE=0.384); however, full specifications of the imaging lens are not provided by the manufacturer, precluding a more accurate estimation of the true diffraction-limited performance. The lens’ NA was estimated to be 0.004 based on the slanted-edge measurement. The measured MTF cutoff frequency matched the USAF target measurement where group 3 to 6 (14.25  lp/mm) was resolvable. As shown in the dermal images, both dermascopes demonstrated sufficient image quality for most reasonably sized lesions, with the ability to resolve features as small as 17  μm for the smartphone dermascope and 70  μm for the USB dermascope.

Illumination uniformity was greater than 85% for all wavelengths with both dermascopes and was easily corrected in the image processing algorithms.

### 5.3 Next Steps

A number of improvements could be made to the systems before conducting a large-scale clinical trial. Currently, the system processing does not incorporate color-to-color spatial image registration. The effects of this are most readily seen in Fig. 7 where the border markings do not completely overlap. Image capture of a full dataset takes about 20 s. Increasing capture speed would reduce the likelihood for image blur between images, easing the need for color-to-color image registration while faster image capture would also increase patient comfort. If image capture speed is not able to be increased, having the clinician deliberately add the markings would likely improve registration because they provide high contrast, well-defined features to extract.

The USB dermascope could benefit from an improved lens design. Future systems could better take advantage of smartphones with two rear cameras and add stereoscopic 3D imaging to its analyses to provide a topography of the skin lesion. Alternatively, the dual cameras could provide two FOVs or two NAs for imaging flexibility.

Additional illumination optics, such as diffusers,^24^ could increase illumination uniformity. The LED board was originally designed to take advantage of the dual cameras of the LG G5, but reducing the center aperture of the LED board could increase illumination uniformity and reduce system size. LED wavelengths could also be better tailored to the task or expanded into UV wavelengths to probe potential autofluorescence signatures.

Finally, to determine the effect of the IR filter on the mapping performance, an additional dermascope should be built and tested with the USB camera in which the IR filter is not removed.

## 6 Conclusion

Two geometries of smartphone-based dermascopes for dermal lesion screening and erythema monitoring using PMSI and PWLI are described. These devices augment the capabilities of PCPs, with the potential for earlier detection of melanoma and NMSC along with quantitative monitoring of erythema. The combination of LED sources, 3D-printing, and smartphone-based imaging enables the creation of low-cost (a high-volume BOM cost of <$40 excluding the smartphone should be easily achievable), feature-rich, easy-to-use medical imaging devices using either the smartphone camera or a USB camera. While initial results are promising, a longitudinal clinical trial along with histopathology gold-standards will be necessary to validate the diagnostic performance of the devices across multiple lesion types and skin types.

## 7 Appendix A: Processing Algorithms Visualized

**Fig. 12:**
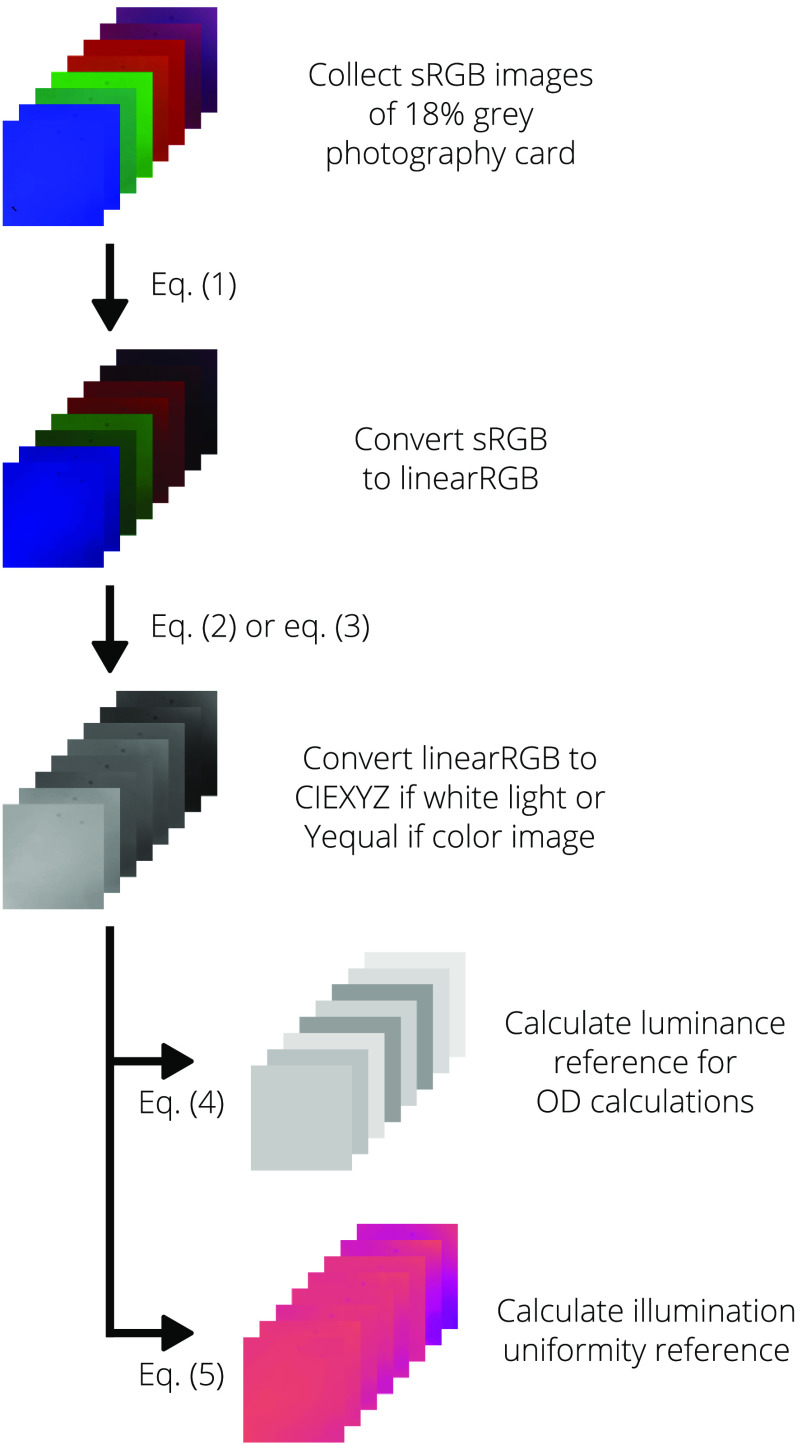
Visual process flow for the reference images as provided in Algorithm 1

**Fig. 13:**
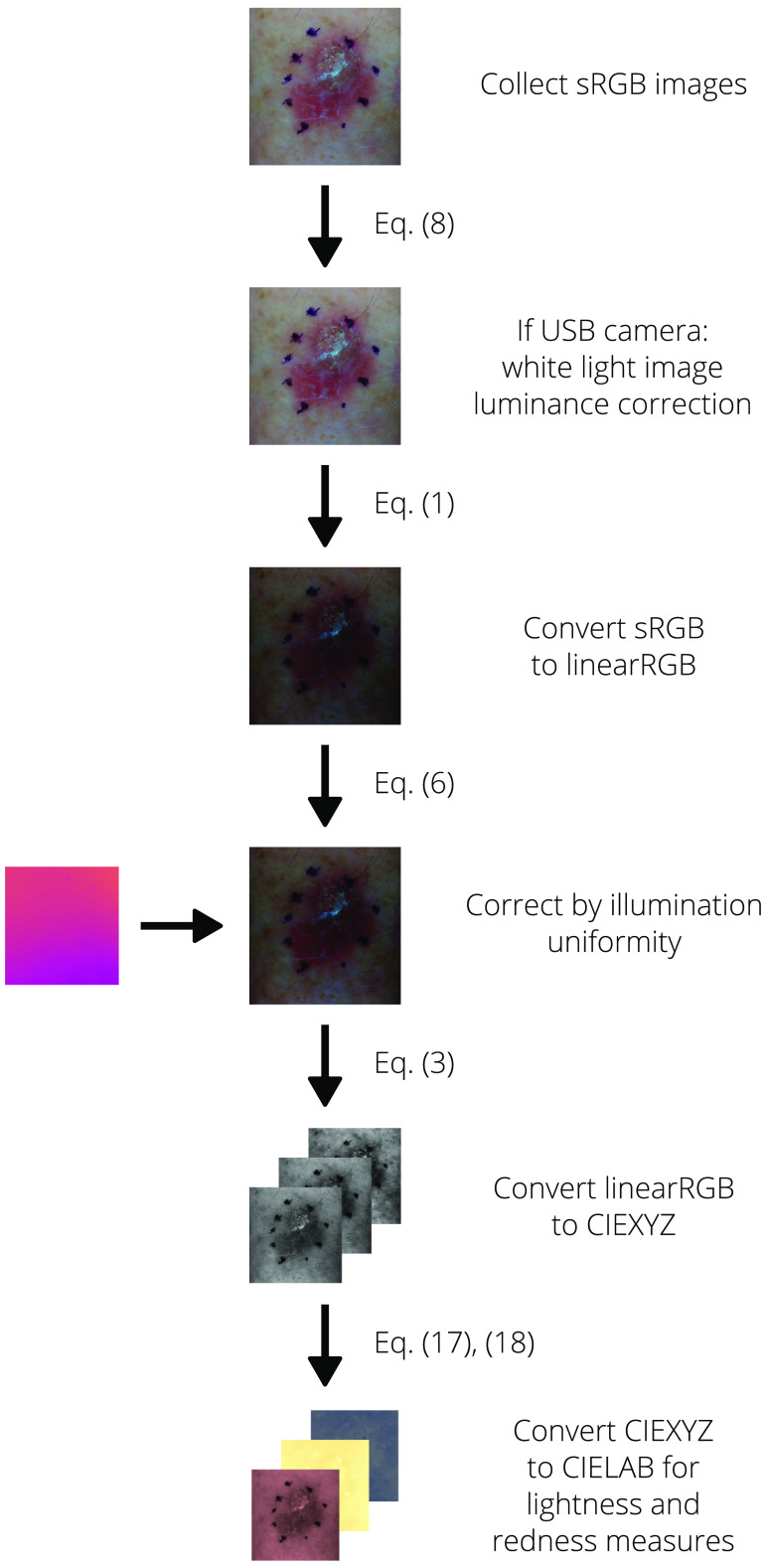
Visual process flow for the white-light images as provided in Algorithm 2

**Fig. 14:**
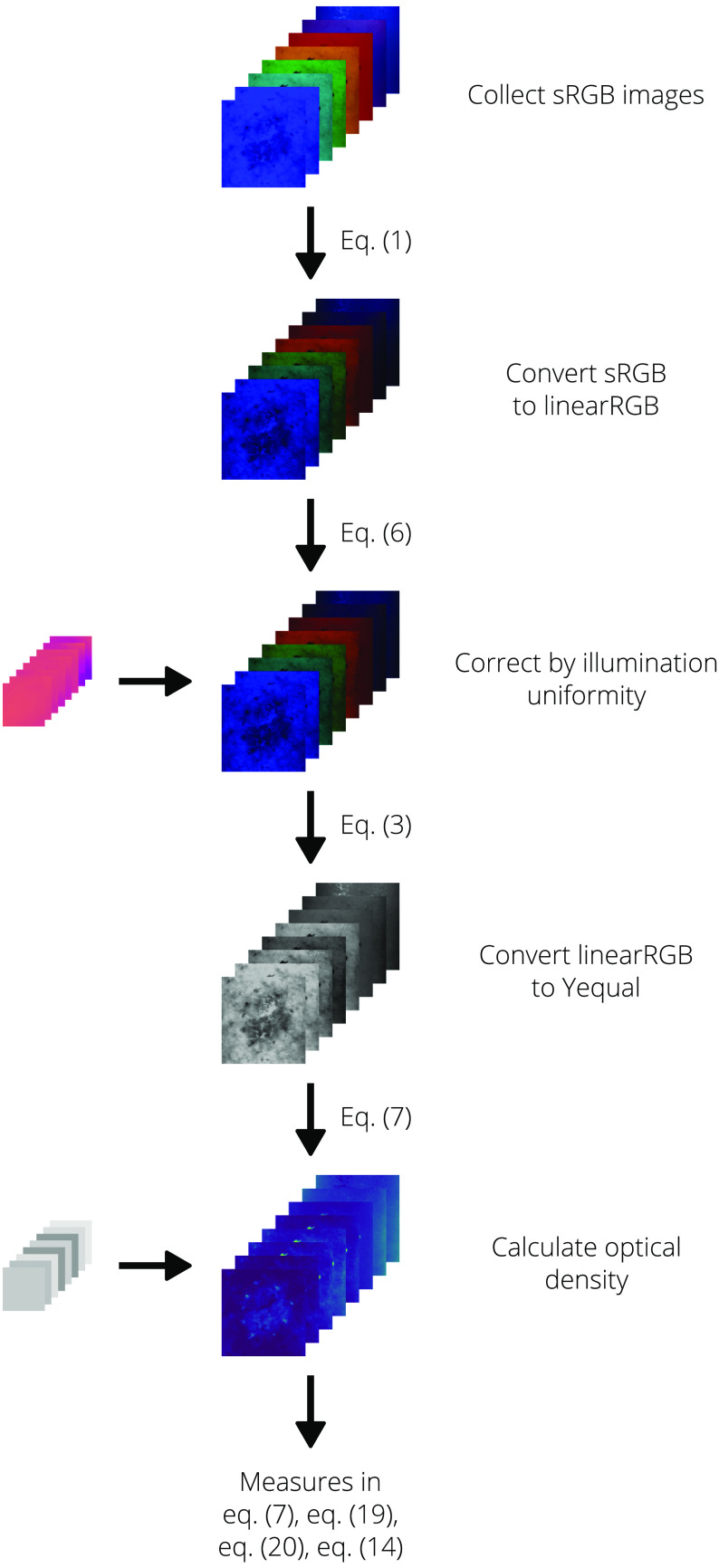
Visual process flow for the multispectral images as provided in Algorithm 2.

Figures 12–14 provide flowcharts to help visualize the processing algorithms for the reference, white-light images, and color images provided in Algorithms 1 and 2.

## 8 Appendix B: Finger Occlusion

**Fig. 15:**
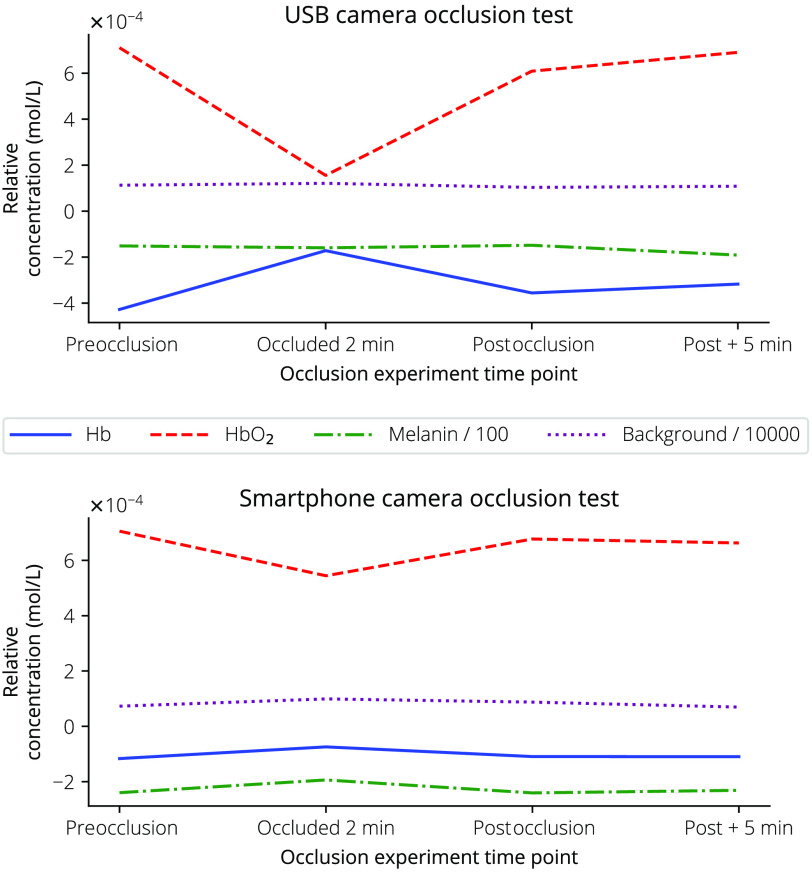
Finger occlusion test results for the smartphone camera and USB camera at preocclusion, after occlusion for 2 min, and postocclusion for Hb, ${\mathrm{HbO}}_{2}$, melanin, and background measures resulting from Eq. (14). Here, the melanin measure has been divided by 100 and the background measure divided by 10,000 for easier comparison with the changes in Hb and ${\mathrm{HbO}}_{2}$.

While the Hb and ${\mathrm{HbO}}_{2}$ chromophore levels should change during the finger occlusion test as shown in Fig. 8, the melanin and background measures should remain constant. Figure 15 shows the additional measures during the occlusion test. Here, the melanin measure has been divided by 100 and the background measure divided by 10,000 for easier comparison of changes between measures. Here, the USB camera’s melanin and background measurements are more stable over the time points compared with the smartphone camera.
